# ProHeart® 12, a moxidectin extended-release injectable formulation for prevention of heartworm (*Dirofilaria immitis*) disease in dogs in the USA for 12 months

**DOI:** 10.1186/s13071-019-3632-3

**Published:** 2019-07-26

**Authors:** Tom L. McTier, Kristina Kryda, Martha Wachowski, Sean Mahabir, Deborah Ramsey, Doug Rugg, Mark Mazaleski, Carol Therrien, Eric Adams, T. Wolff, Dwight D. Bowman

**Affiliations:** 1 Zoetis, Kalamazoo, MI USA; 2 The Veterinary Consultancy, Woodcliff Lake, NJ USA; 3 Northern Biomedical Research, Spring Lake, MI USA; 4 Covance, Greenfield, IN USA; 5 Cornell University, Ithaca, NY USA

**Keywords:** Moxidectin, *Dirofilaria immitis*, Heartworm, Prevention, Macrocyclic lactone, Extended-release, Laboratory study, Field study, Dog

## Abstract

**Background:**

The efficacy of an extended-release injectable moxidectin (0.5 mg/kg) suspension (ProHeart® 12) (PH 12) in preventing the development of *Dirofilaria immitis* in dogs for 12 months was investigated in laboratory and field studies in the USA.

**Methods:**

In each of two laboratory studies, 20 dogs ≥ 12 months of age were randomly allocated to receive a subcutaneous injection of saline or PH 12 on Day 0 and were then inoculated with 50 *D. immitis* third-stage larvae (L_3_) on Day 365. All dogs were necropsied ~ 5 months post-inoculation for adult worm counts. The field efficacy study included dogs ≥ 10 months of age from 19 veterinary clinics in the USA treated with either 20 monthly doses of Heartgard® Plus (HG Plus) (296 dogs) or two doses of PH 12 (297 dogs) on Days 0 and 365. Efficacy was determined on Days 365, 480 and 605 using adult HW antigen and microfilaria testing to assess adult HW infection.

**Results:**

PH 12 was 100% effective in preventing HW disease in all three of these studies. In the laboratory studies, no PH 12-treated dogs had any adult HWs, whereas all control dogs in both studies had adult HWs [geometric mean, 30.2 (range, 22–37) for Study 1 and 32.6 (22–44) for Study 2]. In the field study, all dogs treated with PH 12 tested negative for adult HW infection on all test days (Days, 365, 480 and 605), whereas four dogs receiving HG Plus (positive control) tested positive for HWs during the study (three dogs on Day 365 and one dog on Day 480). All four dogs treated with HG Plus that subsequently tested positive for HWs during the field study were from the lower Mississippi River Valley region, where HW resistance to macrocyclic lactone preventives has been confirmed to occur. PH 12 was significantly better than HG Plus in preventing heartworm disease in the field study (*P *= 0.0367). PH 12 was well-tolerated in both laboratory and field studies.

**Conclusions:**

A single dose of ProHeart® 12 was 100% effective in preventing heartworm disease in dogs for a full year in both laboratory and field studies.

## Background

Canine heartworm (HW) disease caused by *Dirofilaria immitis* has been reported from an increasing number of countries, including the USA and those in western Europe where recent data are available; incidence rates of the disease are on the rise in many of these areas [1–5]. In the USA, the American Heartworm Society (AHS) reported a 21% increase in reported cases of dogs positive for *D. immitis* between 2013 and 2016 [3]. However, these same data suggest only approximately 30% of the 70 million pet dogs in the USA are regularly tested for *D. immitis* infection, leaving a large population of dogs unmonitored. Even more concerning is that up to two-thirds of dogs in the USA are estimated to not receive any HW prevention [4].

Macrocyclic lactone (ML)-based products have been used to safely and effectively prevent HW disease in dogs for more than 30 years [6–12]. At their registration, ivermectin (Heartgard-30®, Merial, Duluth, GA, USA), milbemycin oxime (Interceptor®, Elanco, Greenfield, IN, USA), selamectin (Revolution®/Stronghold®, Zoetis, Parsippany, NJ, USA) and moxidectin (ProHeart, Zoetis, NJ; Advantage Multi/Advocate, Bayer, Shawnee, KS, USA) were all considered 100% efficacious against *D. immitis* when used as per label instructions. These products remain highly efficacious today in the vast majority of cases and regions in the USA when used appropriately [13, 14]. However, there are some serious concerns in general with HW prevention that need to be addressed. Compliance with the AHS/Companion Animal Parasite Council guidelines and recommendations concerning regular testing and year-round monthly preventive administration appears to remain low [4, 14–17]. This low compliance combined with the upward trend in incidence of canine HW infection and confirmed reports of emerging ML resistance [18–23] to current preventive products present an evolving and complex problem that requires innovative solutions.

ProHeart® 6 (PH 6) (Zoetis, Parsippany, NJ, USA) was developed with the specific goal of improving owner compliance with canine HW disease prevention. In contrast to other HW preventive products, PH 6 uses a unique microsphere technology that provides extended release of moxidectin. Most of the monthly-administered ML-based products (ivermectin, milbemycin oxime and selamectin) do not provide continuous protection against HW infection. These molecules are relatively short-lived and likely are not active against all incoming HW larvae as they are transmitted; they work primarily by killing the larvae acquired within the previous 30 days. PH 6 delivers preventive levels of moxidectin in a continuous manner, exposing incoming larvae to the active moxidectin at the time of transmission. In fact, a single dose of PH 6 (0.17 mg/kg) protects dogs against HW disease for 6 months [10, 11]. This long-lasting activity enables owners to reliably protect their dogs with twice-yearly dosing requirements. ProHeart® 12 (PH 12) (Zoetis, Parsippany, NJ, USA) uses the same microsphere technology as PH 6 but with a higher final concentration of microspheres, providing 3× (0.5 mg/kg moxidectin) the dose rate of PH 6. The identical product is already registered in Australia, New Zealand and Japan as ProHeart® SR-12 (PH SR-12). PH SR-12 has been marketed in Australia for more than 18 years and is the leading heartworm preventive product in this country. A single dose has been shown to protect dogs against HW disease for an entire year, reducing owner compliance requirements to a single annual treatment [24]. Field studies were conducted in Australia, Japan and in the USA (2002–2003, prior to the present field study in the USA) using PH SR-12, all indicating 100% efficacy, but these study results were never published (Zoetis data on file). Collectively, these data have demonstrated the robust preventive efficacy of PH SR-12 in these countries. An additional benefit of the injectable PH 6 and PH 12 formulations is that administration occurs within the setting of a veterinary clinic, promoting dosing monitoring, opportunities for HW testing and providing reliable data regarding an animal’s preventive regime compliance.

The purpose of the present studies was to investigate the efficacy of an extended-release injectable moxidectin (0.5 mg/kg) suspension (PH 12) in preventing the development of *D. immitis* in dogs for 12 months in (i) two laboratory studies using US isolates recently collected from the field; and (ii) in a multi-site clinical field study where dogs were under natural exposure to HWs across multiple regions of the USA.

## Methods

### Study guidelines and masking

The two laboratory studies were masked, negative placebo-controlled, randomized efficacy studies. Study 1 was conducted at Northern Biomedical Research, Inc. (Spring Lake, MI, USA), and Study 2 was conducted at Covance Laboratories, Inc. (Greenfield, IN, USA). Procedures used in the studies were carried out in accordance with the CVM Guidance for Industry #90, Efficacy Requirements for Anthelmintics: Overall Guidelines (VICH guideline GL7) [25] and CVM Guidance for Industry #111, Effectiveness of Anthelmintics-Specific Recommendations for Canine (GL19) [26]. Personnel administering treatment (other than owners) or conducting *D. immitis* inoculations, running antigen or microfilaria tests, conducting adult *D. immitis* counts, making animal observations or performing general care of dogs were always masked to treatment allocation.

### Laboratory efficacy studies

#### Animals

Individually identified, purpose-bred intact male and female beagles ≥ 12 months of age were used in both laboratory studies. Dogs were sourced from Ridglan Farms (Mt. Horeb, WI, USA) for Study 1 and from Covance Laboratories Inc. (Cumberland, VA, USA) for Study 2. All animals received a physical examination by a veterinarian to determine health and suitability for inclusion in the study. Weighing on Day-2 (Study 1) or Day-4 (Study 2) confirmed all dogs were between 6.7 and 12.5 kg at the time of study initiation. Animals were acclimated for at least 14 days prior to treatment.

All dogs were housed individually within a mosquito-proof facility in indoor cages that conformed to accepted animal welfare guidelines. They were fed an appropriate maintenance diet of a commercial dry canine ration and had access to water *ad libitum*. Standard accepted environmental conditions were maintained, and environmental enrichment and social interactions were provided. Twenty dogs were used in each study, and each treatment group (*n *= 10) consisted of approximately equal numbers of male and female dogs.

#### Design

No dog was treated with an avermectin within 90 days prior to the start of the study (Day 0). Dogs were determined to be free of HW infection prior to treatment *via* testing on Day-19 (Study 1) or -14 (Study 2) and again on Day 240 (Studies 1 and 2). The absence of circulating microfilariae was verified using a modified Knott’s test, and commercially available tests (SNAP® HTWM, Idexx Laboratories, Westbrook, ME, USA or DiroCHEK®, Zoetis, Parsippany, NJ, USA) were used to confirm a lack of circulating adult *D. immitis* antigen. Serological tests were repeated on Day 485 (both studies) and on Day 516 (Study 1) or Day 518 (Study 2) to potentially identify infections from the experimental inoculation on Day 365.

On Day-2 (Study 1) or Day-4 (Study 2), 20 dogs were allocated randomly to treatments and to pens according to a randomized complete block design with a one-way treatment structure. Blocking was based on pre-treatment body weight (Day-2, Study 1; Day-4, Study 2) and pen location. Each study contained two treatment groups: T01, negative control and T02, PH 12 on Day 0. All dogs were inoculated with 50 *D. immitis* third-stage larvae (L_3_) on Day 365. Preventive efficacy was evaluated at ~ 5 months post-inoculation (PI) [Day 517 (Study 1) or 519 (Study 2)] following necropsy and adult worm recovery and enumeration.

#### Treatment

For both studies, dogs were treated on Day 0 with either the control (saline, T01) or test (PH 12, T02) formulation *via* sub-cutaneous injection in the left or right side of the dorsum of the neck cranial to the scapula. The test product was prepared as an injectable suspension of 10% w/v moxidectin microspheres in sterile vehicle, and all treatments were administered using a 1-ml syringe. The product was administered at a volumetric ratio of 0.05 ml/kg to deliver a dose of 0.5 mg/kg body weight. The negative control treatment consisted of sterile saline administered at 0.05 or 0.5 ml/kg. All doses were calculated according to body weights of dogs collected on Day-2 (Study 1) or Day-4 (Study 2).

#### Animal observations

Clinical observations were made for all dogs on the day of treatment (Day 0) prior to dosing and then at 1, 3, 6 and 24 h post-treatment. Injection sites were evaluated at times of clinical observations. General health observations of all dogs were conducted by appropriately trained personnel twice daily throughout the duration of the study, with the following exceptions: health observations were not made on Day 0 and were made only once on Day 1, as multiple other clinical observations were performed within this 24-h period. Health observations were also made only once on the day of necropsy. Dogs were given physical examinations prior to treatment (Study 1, Day-21; Study 2, Day-14), on Days 181 and 365 and again within 48 h of necropsy (Study 1, Day 516; Study 2, Day 517). Examinations included, but were not limited to, rectal temperature, thoracic auscultation, skin and hair coat assessment, and an assessment of the general physical condition of each dog. Dogs were weighed for dosing calculations on Day-2 (Study 1) or Day-4 (Study 2), again on Days 181 and 365, and within 24 h of necropsy (Study 1, Day 516; Study 2, Day 519) as part of the health monitoring protocols. Study 1 also included the recording of additional weights approximately every 2 weeks. For both studies, all personnel conducting observations were masked to treatment allocations.

#### Inoculation with *D. immitis*

On Day 365 (365 days post-treatment) of each study, each dog was inoculated with 50 *D. immitis* third-stage (L_3_) larvae in RPMI media by subcutaneous injection in the right inguinal region. Larvae were harvested from infected *Aedes aegypti* mosquitoes reared and maintained at Zoetis (Kalamazoo, MI, USA) as previously described [27].

The heartworm strains used in these studies were from isolates collected from naturally infected dogs from the southeastern USA within 3 years of the study start. Study 1 used the *D. immitis* strain ZoeAL-01-2015 (ZoeAL), which was originally collected from a dog from Wetumpka, AL in March 2015. Study 2 used the *D. immitis* strain GCFL-01-2014 (GCFL), obtained from a 3-year-old mixed breed from the Fort Myers, FL area in September 2014.

#### Adult *D. immitis* counts

Dogs were humanely euthanized following an intravenous injection of heparin using an approved euthanasia solution (Fatal-Plus®, Vortech Pharmaceuticals, Ltd., Dearborn, MI, USA) on Day 517 (Study 1) or Day 519 (Study 2). The pleural and peritoneal cavities were examined for adult *D. immitis*, the posterior and anterior vena cavae were clamped, and the heart and lungs were removed. The precava, right atrium, right ventricle and pulmonary arteries (including those coursing through the lungs) were dissected and examined. Any adult worms present were recovered and classified as male or female and as either dead (worms abnormal in both appearance and motility) or alive (all other worms), according to Holmes et al. [28]. Dogs were randomly assigned to order of euthanasia and necropsy.

#### Data analysis

The dog was the experimental unit for treatment. The numbers of adult *D. immitis* worms recovered during *post-mortem* examination were summarized for each treatment group. The natural [log_e_ (x + 1)] transformation was applied to all counts prior to analysis, and the geometric means (back-transformed least squares means) were calculated.

Percent efficacy, relative to the control group and based on geometric means, was calculated as follows:

$$\% Efficacy = \frac{{\left( {Mean\;Control - Mean\;Treated} \right)}}{Mean\;Control} \times 100$$

Treatments with prevention rates of 100% were considered efficacious. Treatment differences were assessed between control and treated groups using contrasts in a general linear mixed model analysis of natural logarithm transformed worm counts and a two-sided significance level of *α *= 0.05. All analyses were carried out using SAS/STAT Release 9.4 (SAS Institute, Cary, NC, USA).

### Multi-center field efficacy and safety study

#### Animals

Dogs (*n *= 593) were recruited from 19 veterinary hospitals across a geographically diverse region of the USA, including a larger number from the southeast where HW prevalence is known to be higher. Enrolled dogs were male or female (intact or neutered), ≥ 10 months of age and ≥ 2.5 kg on Day 0, healthy, not pregnant or lactating (or intended for breeding), client-owned animals confirmed negative for HW at the start of the study (Day-3 to Day 0). A dog with a pre-existing condition could be included if under stable veterinary management. Dogs came from single-dog households and households with other dogs and/or cats, although only one dog per household was enrolled. Diverse households were represented in the sample, with some dogs living indoors or outdoors only and others living both indoors and outdoors. Dogs were excluded if they were sick, debilitated or had a history of apparent reactions to either the test or control product or had received PH 6 within 12 months prior to Day 0. Dogs reported to be on other HW preventive were excluded if they had been treated within the time limit specified on the product label prior to the initial screening visit (Day-3 to Day 0). All dogs were housed and maintained under their normal home conditions for the duration of the study. Animal health was monitored daily by the owner and at scheduled clinic visits by qualified veterinary personnel. No additional products with efficacy against *D. immitis* larvae, adults or microfilariae were permitted to be used on any enrolled animals.

#### Design

The study was conducted as a randomized, single-masked, multi-centered clinical study with a positive control. A site was required to enroll a minimum of two evaluable cases per treatment group to participate, and no single site was permitted to enroll more than 40% of the total evaluable cases included in the study. Dogs were enrolled in order of presentation to the veterinary practice, if they met all inclusion and no exclusion criteria. In households with multiple dogs, the study-eligible dog was the first dog presented that fulfilled enrollment criteria. Within each clinic, dogs were blocked in sets of two; within each block, the dogs were randomly allocated to either the positive control (T01, n = 296) or test treatment (PH 12; T02, n = 297) group.

Eligibility for inclusion in the study was determined at the initial screening visit, between Days-3 and Day 0. At this time, each dog’s details and history were recorded, and all dogs were given a physical examination (including body weight and temperature) to assess general health. Blood was also collected to test for *D. immitis* infection. Dogs enrolled in T01 received Heartgard® Plus (HG Plus) monthly for 20 months, and dogs enrolled in T02 received PH 12 (0.5 mg/kg) on Days 0 and 365 (± 3 days for each injection). All dogs were required to return to the clinic on Days 120, 240, 365, 480 and 605 (± 3 days for all visits) for monitoring, treatment and HW testing. Efficacy of PH 12 was determined via testing for HW infection on Days 365, 480 and 605 using adult HW antigen tests and the modified Knott’s test for microfilariae. Dogs with confirmed positive test results for HW on a given day were removed from the study and processed for adult HW treatment. The Veterinary Consultancy (Woodcliff Lake, NJ, USA) provided contract resources for the execution of this study.

#### Treatment

All dogs received one of two forms of HW preventive for the duration of the study. Dogs in the positive control group (T01) were administered HG Plus (ivermectin + pyrantel) chewable monthly by their owners either at the clinic on scheduled visit days (Days 0, 120, 240, 365 and 480 ± 3 days for each visit) or at home (Days 30, 60, 90, 150, 180, 210, 270, 300, 330, 390, 420, 450, 510, 540 and 570 ± 3 days for all doses). All of the HG Plus product was supplied by the veterinary clinic based on the most recent body weight of the dog and the dosage chart for HG Plus, such that the minimum recommended dose of 6 µg/kg ivermectin was achieved. Sufficient product was given to each owner to ensure monthly treatment between scheduled clinic visit days. Dogs assigned to the test group (T02) were administered a subcutaneous injection of PH 12 by a certified and registered user of PH 6 at the veterinary clinic on Days 0 and 365 (± 3 days). Active product was prepared as an injectable suspension of 10% w/v moxidectin microspheres in sterile vehicle, and all treatments were administered using the smallest size syringe appropriate for the calculated volume. The treatment suspension was formulated at 10 mg/ml moxidectin, and the product was administered at 0.5 mg/kg body weight (0.05 ml/kg).

#### Heartworm testing

Whole blood was collected from all dogs at the initial screening visit and again during scheduled clinic visits on Days 120, 240, 365, 480 and 605 (± 3 days for each visit) for the detection of adult HW infection. To determine initial suitability for the study, blood collected at the screening visit was tested for adult *D. immitis* antigen in the clinic using a commercial HW antigen detection kit following the manufacturer’s instructions. The specific adult heartworm antigen test used was left to the individual clinic’s discretion. The remainder of the screening sample and all additional samples collected during the study were sent to a contract diagnostic laboratory (Marshfield Labs, Marshfield, WI, USA) for additional evaluation. All samples were tested for adult HW antigen using the commercially available HW antigen detection kit, (DiroCHEK®; Zoetis, Parsippany, NJ, USA) in accordance with the manufacturer’s instructions and for *D. immitis* microfilariae using a modified Knott’s technique or membrane filter.

#### Clinical pathology: complete blood count (CBC), clinical chemistry and urinalysis

Samples were collected on Day-3 to 0 and on Day 605 (± 3) for hematology, serum chemistry and urinalysis.

The following analytes of the CBC were summarized: lymphocytes, neutrophils, basophils, eosinophils, hematocrit, hemoglobin, mean corpuscular hemoglobin, mean corpuscular hemoglobin concentration, mean corpuscular volume, monocytes, nucleated red blood cells, platelets, red blood cells, reticulocytes, segmented neutrophils, white blood cells.

The following analytes of the serum chemistry profile were summarized: alanine amino transferase, albumin, albumin/globulin ratio, alkaline phosphatase, amylase, anion gap, aspartate amino transferase, bicarbonate, blood urea nitrogen (BUN), BUN/creatinine ratio, calcium, chloride, cholesterol, creatinine kinase, creatinine, gamma glutamyl transferase, globulin, glucose, lipase, magnesium, phosphorus, potassium, sodium, sodium/potassium ratio, total bilirubin, total protein, triglycerides.

Urinalysis results reported for each animal included the following: urine specific gravity, urine PH, amorphous crystals, bacteria, casts (course, fine, granular, hyaline), crystals, mucous, red blood cells, epithelial cells (renal, squamous, transitional), urine bilirubin, urine color, urine glucose, urine hemoglobin, urine ketones, urine protein, turbidity, urobilinogen, white blood cells.

#### Data analysis

HW antigen and microfilariae test results were summarized in two-way frequency tables by treatment and time point (Table 3). The PH 12 was considered effective if all dogs in the test group had negative HW results at Day 365, 480 and 605. StatXact 10 software was used to test for non-inferiority of PH 12 relative to HG Plus and also to test for a difference between the two groups for the prevention of heartworm infection, based on whether dogs were ever positive for heartworm on Days 365, 480 or 605. Non-inferiority was tested at the one-sided 0.025 significance level using an equivalence margin of 5% using a one-sided 97.5% exact confidence limit for two binomial proportions. A 95% confidence interval for comparing the difference between two binomial proportions based on the standardized statistic and inverting a 2-sided test was used to compare prevention rates.

## Results

### Laboratory efficacy studies

#### Detection of *D. immitis* adult heartworm antigen and microfilariae

No dog in either T01 (Control) or T02 (PH 12) was positive for *D. immitis* microfilariae at any time during the two studies. This is unsurprising as dogs were inoculated on Day 365 and thus would not be expected to be microfilaremic until at least ~ Day 545 (~ 6 months PI). However, adult antigen tests can detect HW infection earlier than this (~ 5 months PI), especially in dogs with many female worms. As expected, all dogs tested negative for *D. immitis* antigen on Day-19 (Study 1) or -14 (Study 2) and on Days 240 and 485 (Studies 1 and 2). Samples collected prior to necropsy (Day 516, Study 1; Day 518, Study 2) from PH 12-treated dogs (both studies) remained negative for *D. immitis* antigen. However, 70% and 60%, respectively, of the control dogs in Studies 1 and 2 were antigen-positive at this time (~ 5 months PI).

#### Heartworm counts

All control (placebo) dogs in both studies had live adult *D. immitis* worms at necropsy. Geometric mean (range) worm counts for control dogs (T01) were 30.2 (22–37) for Study 1 and 32.6 (22–44) for Study 2, confirming the robustness and viability of both *D. immitis* strains used (Table 1). In contrast, all dogs treated with PH 12 (T02, both studies) were negative for adult *D. immitis* worms at necropsy, confirming the 100% effectiveness of PH 12 in preventing the development of *D. immitis* when dogs were inoculated with infective larvae 12 months after treatment. On Study 2, one dog treated with PH 12 was excluded from the study due to incomplete dosing, which was identified at necropsy when this dog was found to have a similar number of worms (12 males and 18 females) to dogs in the control group. Plasma samples collected at necropsy showed this one infected dog in the PH 12-treated group did not have detectable levels of moxidectin, corresponding to the results obtained for the 10 control (placebo) dogs. These results indicated that this one dog in the PH 12 group did not receive moxidectin on Day 0 as intended; therefore, this dog was excluded from the data analysis.

**Table 1:** Laboratory study design and efficacy of ProHeart® 12 against *Dirofilaria immitis* in dogs

Group^a^ (*n *= 10)	Treatment	Dosage (mg/kg)	Day of treatment	No. of dogs with worms	Adult *D. immitis* worm counts^b^		
					Individual worm counts	Geometric mean	Percentage reduction
Study 1							
T01	Vehicle	0	0	10	22, 25, 29, 30, 30, 31, 32, 34, 35, 37	30.2	–
T02	ProHeart® 12 (moxidectin)	0.5	0	0	0	0^c^	100
Study 2							
T01	Vehicle	0	0	10	22, 28, 30, 32, 32, 33, 34, 36, 40, 44	32.6	–
T02^d^	ProHeart® 12 (moxidectin)	0.5	0	0	0	0^e^	100

^a^All dogs were inoculated with 50 *D. immitis* L_3_ on Day 365. Study 1, *D. immitis* ZoeAL-01-2015; Study 2, *D. immitis* GCFL-01-2014

^b^All dogs were necropsied for recovery and enumeration of adult HWs on Day 517 (Study 1) or Day 519 (Study 2)

^c^Geometric mean is statistically different from that of Group T01 (*P* < 0.0001)

^d^*n *= 9 for Group T02 in Study 2; one dog was excluded from study due to incomplete dosing. *n *= 10 for all other groups

^e^Geometric mean is statistically different from that of Group T01 (*P* < 0.0112)

#### Health observations

There were no mortalities among the dogs involved in these studies. No cosmetic changes were observed at the injection sites of any dogs during Study 1. Two control dogs (T01) and one PH 12-treated dog (T02) in Study 2 showed mild and transient changes (mild subcutaneous swelling or mild redness) at the injection site, which were resolved at 24 and 72 h, respectively, for the two dogs in T01, and at Day 30 for the third dog (T02). Various abnormal health observations were made during these studies across all treatment groups. In Study 1, gastrointestinal clinical signs including loose and mucous stool, diarrhea, bloody diarrhea, melena and vomiting were observed in multiple dogs beginning prior to any treatments and continuing throughout the study. Among the 5 dogs most affected (> 30 events during the treatment phase), 3 were allocated to T01 (saline) and 2 were allocated to T02 (PH 12). All other dogs on the study also exhibited gastrointestinal associated clinical signs less frequently. Fecal examination for parasites and/or giardia ELISA tests were negative. Treatment of all 20 dogs with a 5-day course of metronidazole, probiotics, and fiber supplements was ameliorative. A case of chronic dermatitis and one of prostatitis affected dogs in T01 (saline). In Study 2, clinical signs including interdigital cysts with associated lameness, mucoid stool, vomiting and diarrhea, abrasions and scabs associated with rubbing on edges of their cages, and behavioral issues were observed in both treatment groups at about the same distribution and frequency. One dog in T02 (PH 12) had recurrent bouts of soft stools and diarrhea. Fecal examination for parasites, complete blood count, and blood chemistry results were normal. The signs were considered nonspecific and unrelated to treatment.

### Multi-center field efficacy study

#### Animal details

A total of 593 dogs were enrolled in the study, and demographics of the dogs in each group are presented in Table 2. Approximately equal numbers of dogs (297 for PH 12 and 296 for HG Plus) were enrolled in each treatment group, with slightly more females than males in each treatment group. At the time of enrollment, dogs ranged in age from 10 months to 14 years, with a mean age (standard deviation) of 4.7 (2.73) and 4.8 (2.91) years for T01 (HG Plus) and T02 (PH 12), respectively. Seventeen percent of enrolled dogs had or were being treated for a recent/recurring disease, and 35.5% were receiving therapeutic or prophylactic medication. A larger number of pure-bred (320) than mixed-breed (274) dogs were enrolled in the study, with a similar breakdown between the two groups (T01, 52.9% pure-bred; T02, 54.9% pure-bred). The most common breeds enrolled included Labrador Retrievers (41), Golden Retrievers (18), Dachshunds (15), Shih Tzus (15), German Shepherds (14), Chihuahuas (14), Yorkshire Terriers (14), Pit Bull Terriers (12) and English Bull Dogs (10). Where dogs spent most of their time (mostly indoor/mostly outdoor/indoor and outdoor) was similar within each treatment group. Based on the owners’ assessments, 4.9% of the enrolled dogs spent most of their time outdoors, 36.9% indoors and outdoors and the majority of dogs (58.2%) spent most of their time indoors. Most dogs enrolled in the study had received prior treatment with a HW preventive, although 0.7% had never received treatment and 8.3% had received irregular treatment. The preventives listed by owners included topical, oral and injectable products, with more than 50% of owners using either HG/HG Plus or Trifexis® (Elanco, Greenfield, IN, USA).

**Table 2:** Details of dogs in field study investigating the efficacy of ProHeart® 12 against *Dirofilaria immitis*

Category	Treatment group^a^		
	T01	T02	Total
	Heartgard® Plus	ProHeart® 12	
No. of females (spayed/intact)	152 (143/9)	163 (149/14)	315 (53.0%)
No. of males (neutered/intact)	145 (120/25)	134 (107/27)	279 (47.0%)
Initial age in years (range)	4.7 (1.0–13.0)	4.9 (0.8–14.0)	4.8 (0.8–14)
Recent/recurring disease, *n* (%)	55 (18.5)	46 (15.5)	101 (17.0)
Receiving therapeutics/prophylactics for recurrent disease, *n* (%)	107 (36.0)	104 (35.0)	211 (35.5)
Pure-bred/mixed breed (%)	52.9/47.1	54.9/45.1	53.9/46.1
Time spent, *n* (%)			
Indoors and outdoors	106 (35.7)	113 (38.0)	219 (36.9)
Mostly indoors	176 (59.3)	170 (57.2)	346 (58.2)
Mostly outdoors	15 (5.1)	14 (4.7)	29 (4.9)

^a^*n *= 296 for Heartgard® Plus and 297 for ProHeart® 12

Ninety animals (51, HG Plus; 39, PH 12) were withdrawn from the study prior to the final visit on Day 605. For more than half of the dogs (48/90, 53.3%), the reason for withdrawal was due to owner relocation, rehoming of the dog or non-compliance by the owner. Other reasons included death unrelated to treatment (15; 8 HG Plus and 7 PH 12), euthanasia (8; 2 HG Plus and 6 PH 12), other medical conditions unrelated to the study (4; 3 HG Plus and 1 PH 12), prohibited drug administration (2 HG Plus) and possible drug reaction (1 dog from each treatment group). Six dogs (three from each treatment group) were removed from the study after their Day 0 blood tests were positive for *D. immitis*. Four dogs in the HG Plus group that became HW-positive during the study were also removed from the study upon confirmation of their HW-positive status.

#### Efficacy

All animals completing the 605-day study received either 20 doses of HG Plus (T01) or two doses of PH 12 (T02), resulting in a final total of 218 evaluable cases for HG Plus and 236 evaluable cases for PH 12 across evaluable cases Days 365, 480 and 605.

Four dogs treated with the positive control product (HG Plus) were removed from the study after testing positive for adult HW infection on study Day 365 (three dogs) or 480 (one dog) (Table 3). Three of the four dogs tested positive for both adult *D. immitis* antigen and microfilariae; the fourth dog was negative for microfilariae but positive on four adult antigen detection tests (using tests from two different manufacturers), suggesting the presence of a single-sex female worm infection or pre-patent infection. Two of the HG Plus dogs that tested positive for adult HW infection were from the same clinic in Zachary, LA, one was from a clinic in Lake Charles, LA and one was from a clinic in Memphis, TN. All remaining HG Plus dogs tested on Day 605 were negative for adult *D. immitis* infection. The preventive efficacy for HG Plus was 98.2%.

**Table 3:** Results from clinical field study on efficacy of ProHeart® 12 against *Dirofilaria immitis* in dogs

Treatment group	Study day	Adult HW antigen test				Microfilaria test				Total no. of dogs	Prevention rate (Day 365)	Prevention rate (Day 480)	Prevention rate (Day 605)	Overall prevention rate
		Negative		Positive		Negative		Positive						
		No. of dogs	%	No. of dogs	%	No. of dogs	%	No. of dogs	%					
Heartgard® Plus (T01)	365	215	98.6	3	1.4	216	99.1	2	0.9	218^a^	98.6% (215 of 218)	99.5% (208 of 209)	100% (201 of 201)	98.2% (214 of 218)
	480	208	99.5	1	0.5	208	99.5	1	0.5	209^b^				
	605	201	100	0	0	201	100	0	0	201				
ProHeart® 12 (T02)	365	235	100	0	0	235	100	0	0	235	100% (235 of 235)	100% (226 of 226)	100 (222 of 222)	100%^c^ (235 of 235)
	480	226	100	0	0	226	100	0	0	226				
	605	222	100	0	0	222	100	0	0	222				

^a^Three dogs (treated with Heartgard® Plus) were withdrawn after testing positive for adult HW antigen and/or microfilariae on Day 365

^b^One dog (treated with Heartgard® Plus) was withdrawn after testing positive for adult HW antigen and/or microfilariae on Day 480

^c^ProHeart® 12 significantly better than Heartgard® Plus (*P *= 0.0367)

No dogs treated with PH 12 tested positive for adult HW infection on any of the study test days (Day 365, 480 or 605), indicating a 100% prevention of HW disease in all animals for the 12-month period (March–June 2015 to March–June 2016) under test during this study.

Analysis of the field study data for whether an animal was positive for at least one heartworm test over Days 365, 480, and 605 demonstrated that PH 12 was non-inferior to HG Plus for an equivalence margin of 5% at the one-sided 0.025 level of significance (unconditional test of non-inferiority using the difference of two binomial proportions: *t *= − 4.13, *P* < 0.0001), and it also had a significantly higher prevention rate (100% and 98.2% for PH 12 and HG Plus, respectively; 95% CI for difference: 0.2–4.8%; exact test using difference of two binomial proportions based on standardized statistic and inverting a two-sided test, *t *= 2.09, *P *= 0.0367).

#### Health observations

Over the 20-month period of the field study, abnormal clinical signs were recorded in study animals. Most of the dogs experienced at least one abnormal clinical sign, 252 (85.1%; HG Plus) and 261 (87.9%; PH 12) respectively, due to the long study duration. Most of these observed abnormal clinical signs were consistent with sporadic occurrences of conditions commonly observed in the general dog population. The most common abnormal clinical signs occurring in PH 12-treated dogs were vomiting (25.3%), lethargy (15.5%), diarrhea (14.5%) and anorexia (13.8%); incidences of these signs were similar or higher in HG Plus-treated dogs (Table 4). On the day of and the day following dosing, vomiting, lethargy and diarrhea were the most commonly reported signs for both PH 12 and HG Plus-treated dogs. Mild injection site reactions occurred in six PH 12-treated dogs and were observed from one to seven days post dosing; they resolved without any concomitant treatment.

**Table 4:** Adverse reactions reported from field study on efficacy of ProHeart® 12 against *Dirofilaria immitis* in dogs

Adverse reaction^a^	Heartgard® Plus (*N *= 296) *n* (%)	ProHeart® 12 (*N *= 297) *n* (%)
Vomiting	78 (26.4)	75 (25.3)
Lethargy	34 (11.5)	46 (15.5)
Diarrhea	46 (15.5)	43 (14.5)
Anorexia	31 (10.5)	41 (13.8)
Seizures^b^	7 (2.4)	10 (3.4)
Hepatopathy	3 (1.0)	8 (2.7)
Hypersalivation^c^	3 (1.0)	7 (2.4)
Anaphylactoid/hypersensitivity reaction	4 (1.4)	6 (2.0)

^a^Occurrence calculated on a per dog basis. Some dogs may have experienced more than one adverse reaction or more than one occurrence of the same adverse reaction during the study

^b^Seizure category includes all dog reported with epileptic seizures, convulsions and loss of consciousness. Some, but not all, dogs listed with central nervous system disorder not otherwise specified and muscle tremors are also included in this category

^c^Hypersalivation category includes all dogs reported with hypersalivation and gastrointestinal foreign body not otherwise specified

Less frequently, PH 12-treated dogs were also reported with seizures (3.4%), hepatopathy (2.7%), hypersalivation (2.4%) and anaphylactoid/hypersensitivity reaction (2.0%); incidences of these signs were also observed in HG Plus-treated dogs (Table 4). Hypersensitivity-related reactions observed in this field study and across the targeted PH12 safety laboratory studies have been comprehensively summarized and discussed [29]. Of the eight PH 12-treated dogs reported with hepatopathy, two dogs had pre-existing elevation in liver enzymes prior to treatment, one dog experienced elevated liver enzymes during the study that did not appear to be contemporaneous with PH 12 administration and returned to normal by study end, and one dog was noted to have mild elevations in liver enzymes at study end, but the sample was hemolyzed and the investigator did not determine that the other chemistry values were clinically significant. The remaining four dogs had elevations in liver enzymes likely secondary to disease conditions and/or treatment with medications.

During the study, any of the medications that were continued after the dog enrolled on the study were recorded and the safety of concomitant treatment was evaluated as part of the normal observations of the study. Overall, 252 unique medications were administered to dogs (T01 and T02 combined) during the 605-day study.

#### Clinical pathology summary

Clinical pathology summary statistics revealed no effects that appeared clinically significant or biologically important [29]. The mean values for all the hematology and serum chemistry analytes summarized were within the laboratory reference range for each analyte in both treatment groups at both study visits (Day 0 and 605). The urinalysis summaries showed no apparent differences between the HG Plus-treated and PH 12-treated dogs at either time point (Day 0 and 605). The mean and median urine pH of samples collected on Day 0 and 605 were outside the laboratory reference range (5.2–6.8) in both treatment groups. The reason for the overall elevated urine pH in both treatment groups is unknown.

## Discussion

PH 12 (0.5 mg/kg moxidectin in an extended-release injectable suspension) was 100% effective in preventing heartworm disease caused by *D. immitis* in dogs for one year in two separate laboratory studies using two different heartworm strains and in 297 dogs in a large multi-site clinical field study. PH 12 was well tolerated in these three studies. Mild injection site reactions were observed in some animals treated with PH 12, while abnormal clinical signs occurred in similar frequencies across both HG Plus and PH 12 groups in the field study.

As with other MLs used in the prevention of canine HW disease, moxidectin targets developing *D. immitis* larvae. Initial studies using moxidectin in an oral formulation indicated that this molecule is the most potent of the MLs in preventing HW disease in dogs, with a single oral dose as low as 0.5 µg/kg providing 100% protection against developing heartworm larvae when administered 60 days after the inoculation of susceptible infective HW larvae [8]. Subsequent to these findings, moxidectin was developed as an oral preventive and commercialized at a dose of 3 µg/kg (ProHeart® tablets, Zoetis, Parsippany, NJ, USA), which was approved for use in dogs in the USA but was only marketed for a very short time in the early 1990s; it remains on the market in some Asian and Latin American countries.

Moxidectin, like other MLs, has a proven record of effectiveness in preventing *D. immitis* infection in dogs [8, 10–12, 30–33]. However, moxidectin differs from other MLs in that it is highly lipophilic and generally has a longer elimination half-life and larger volume of distribution than ivermectin [34], allowing it to remain in host tissues and subsequently be released into the plasma over time [35]. The microsphere technology used in PH 6 and PH 12/SR-12 maximizes the unique inherent properties of moxidectin, resulting in a prolongation of moxidectin systemic availability and therefore longer-lasting HW protection. This extended protection is in contrast to the shorter protection offered by some MLs, which are eliminated more quickly from the body and require monthly doses to ensure robust prevention [36–38]. Most monthly HW preventives work by killing the larval HWs inhabiting the host at the time of treatment, with little or no residual activity. PH 6 and PH 12 both provide extended protection in dogs against HW disease [10, 11, 24, 39, 40] to provide immediate preventive effects as HW larvae are introduced into the dog due to the persistent availability of the drug in these formulations.

The most common reason for failure of HW preventives is believed to be poor compliance due to incorrect or inconsistent administration, which then allows incoming larval stages of *D. immitis* to mature into adult worms [14–16, 41–43]. Providing guaranteed 12-month compliance together with 12-month heartworm prevention is a significant advance for the USA, especially as owner compliance with monthly administered products in the USA remains far from ideal. According to the American Animal Hospital Association (AAHA) Compliance Follow-Up Study [44], compliance for HW preventive dosing in interviewed owners ranged from 45% in 2003 to 51% in 2009, and these data are reflected in clinical findings across the country [15, 43]. For example, when reviewing lack of efficacy (LOE) data from 271 dogs on HW prevention, Atkins et al. [15] concluded that in more than 80% of the LOE cases insufficient preventive was purchased to provide year-round protection, as recommended by the AHS. This problem was also observed in the field efficacy study reported here, with 9% of enrolled dogs reported to have been inconsistently receiving HW prevention prior to enrollment in the study. A single injection of PH 12 given by a trained professional in a veterinary setting and recorded in the clinic’s records is a reliable means to ensure complete year-round protection of dogs against heartworm disease and provides dog owners with guaranteed compliance upon administration and ensures accurate and complete dosing.

In addition to the increasing incidence of HW infection in recent years, there have been reports of apparent LOE associated with HW preventive products made to the US Food and Drug Administration, Center for Veterinary Medicine (CVM). These LOEs were first identified and reported by Hampshire [45], with subsequent analysis of cases focusing on the lower Mississippi River Valley (LMRV) region, an area that includes large portions of Louisiana, Mississippi and Arkansas, western portions of Tennessee and Kentucky and southern portions of Missouri and Illinois. Currently, analyses to determine patterns within more than 45,000 reported LOE cases from 2004–2015 are ongoing. Though the final data analysis is pending, preliminary findings, while not intentionally regionally focused, indicate a concentration of the LOE cases in the LMRV, with some significant foci identified within that region [46]. It should be emphasized that LOE cases from the LMRV are over-represented in the reported cases, likely at least partially due to the higher transmission rate of HW in this region in addition to non-compliance issues. The contribution of ML resistance to these LOE cases is yet to be determined. However, others have suggested that the increase in HW disease incidence observed in recent years could be due to a confluence of factors, including lack of preventive usage, incorrect administration (dose rate and/or technique) and the emergence of HW resistance [4].

Resistance of some strains of HW to multiple MLs has been confirmed. Indeed, at least 15 different strains/isolates of *D. immitis* resistant to MLs have been identified [18–23]. Most of these strains are from the LMRV of the USA, and investigations on these strains have indicated failure of all current preventive products to provide 100% efficacy in at least one laboratory study with at least one strain.

Injectable PH products have been shown to be active against some *D. immitis* strains classified as ML-resistant. PH 6 (0.17 mg/kg moxidectin) was not effective (21%) against a resistant HW strain (JD-2009) when dogs were inoculated with L_3_ 180 days after treatment with a single dose of PH 6 [19]. However, PH 6 reduced the development of the highly ML-resistant JYD-34 strain by 99.5%, with only a single worm reported from one of six dogs, when dogs were inoculated with L_3_ at the time of dosing [33]. This latter study simulated efficacy against larvae inoculated either at the time of dosing or at the end of the dosing interval (as was done in reference [15] above) when dogs would be re-treated six months after the first treatment. In contrast to the PH 6 results, three consecutive monthly treatments using approved doses of ivermectin, milbemycin oxime and selamectin reduced the development of HWs by ≤ 52% (with all dogs harboring infections) against this strain when treatment was started 30 days PI of L_3_ [21]. Six consecutive monthly treatments with milbemycin oxime, beginning 30 days PI, only increased the efficacy to 72.1% [47]. Additionally, PH 6 and SR-12 demonstrated high (> 95%) microfilaricidal activity against the ML-resistant ZoeMo-2012 strain, which is related to JYD-34 [48]. Additionally, in the field study presented here, no dogs treated with PH 12, including dogs that were from areas where ML resistance has been documented to occur and were under field exposure during a period of likely high infection pressure, developed HW infections.

The laboratory and clinical field studies described herein demonstrate that PH 12 provided dogs with complete 12-month protection against HW disease. All dogs treated with PH 12 in both laboratory studies were found to be free of adult *D. immitis* worms at necropsy. A single dose of the PH 12 extended-release moxidectin injectable suspension at 0.5 mg/kg resulted in 100% prevention of HW development of both the GCFL and ZoeAL strains of *D. immitis*, when inoculated 12 months after treatment. In contrast, all ten control dogs in both laboratory studies developed adult *D. immitis* worms [geometric mean, 30.2 (Study 1) or 32.6 (Study 2)]. These results are similar to those for control dogs from other recent studies [19, 32, 33, 48] and satisfy the VICH guidelines for efficacy [25, 26]. Both of the strains used in these studies were recently (within the previous three years before study initiation) obtained from client-owned animals from the southeastern USA (AL and FL) and were considered representative of the heartworm strains naturally occurring in canine populations from these areas. Both GCFL and ZoeAL strains were obtained directly from the field as microfilariae from naturally infected dogs, passaged through mosquitoes and inoculated into recipient dogs (F1) as third-stage larvae. These original recipient F1 dogs were used as the donor dogs to infect mosquitoes to provide the third-stage larvae to inoculate the animals for these laboratory studies. Both of these strains have been determined to not be resistant to MLs in accordance with Zoetis’ interpretation of the appropriate guidance [25].

**Fig. 1:**
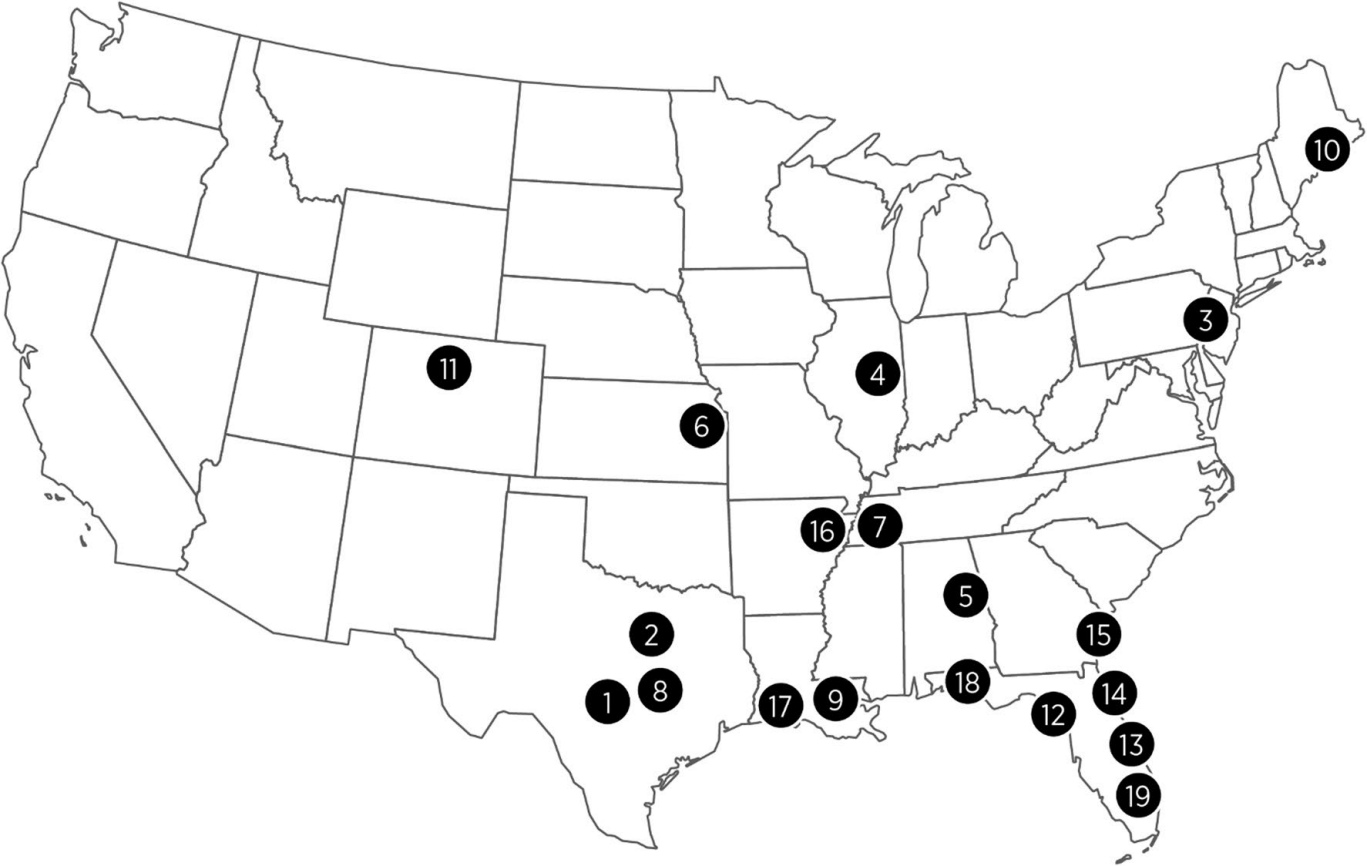
Veterinary clinics in a field study assessing 12-month efficacy of ProHeart® 12 against *Dirofilaria immitis*

In a very large and robust field study assessing natural exposure to heartworms when animals were held in their regular home environments and incorporating 593 client-owned dogs [297 for PH 12 and 296 for the positive control (HG Plus)], PH 12 demonstrated complete prevention of HW disease in dogs for 12 months. One of the common criticisms of the field study design is that exposure of animals to HWs cannot be guaranteed or controlled, as field conditions/environments can vary widely and be influenced by fluctuating climatic conditions from year to year. However, these variables can be somewhat mitigated by a carefully designed study. The opportunity for HW exposure during the field study can be enhanced by placing dogs in areas where, and during the season when, maximum HW transmission is known to occur. Various publications are available to indicate exposure levels to HWs across the USA, and data are available on the actual HW transmission rates in dogs placed outside in the southeastern USA. Dogs placed outdoors in southern Louisiana were infected, with an average of 25 worms in a 12-month period and an average of 6.8 and 5.4 worms in southern GA and central FL, respectively [49]. More recent data reported by Drake & Wiseman [4] using AHS surveys from 2013–2016 suggest the overall incidence of HW disease appears to have increased, particularly in the southeastern USA, during the time that the present study was conducted (2015–2016). In addition, Wang et al. [50] examined the factors influencing the prevalence of HW disease in the USA and produced an algorithm-based map predicting HW prevalence. This map predicts a very high prevalence (5–20%) of HW in the lower mid-west and extending eastward throughout the southeast states. Based on this map, nine of the 19 sites (1, 2, 5, 7, 8, 9, 15, 16 and 17) in our field study were in the area considered to have the highest HW prevalence (5–20%), and five additional sites (6, 12, 13, 14 and 18) were in the area considered to have moderate-high prevalence (2–5%), with site 19 just outside this area (Fig. 1). The majority of evaluable dogs (131 for PH 12 and 125 for HG Plus) on the study were therefore likely exposed to substantial HW challenge during the 12-month evaluation phase (March-June 2015 to March-June 2016) of the PH 12 study. In addition, four dogs treated with HG Plus became infected with HWs during the study, confirming HW transmission, at least in Louisiana and Tennessee, even in the face of well documented monthly preventive therapy with ivermectin. Of all the dogs completing the field study, 101 (22%) evaluable cases were from the LMRV region, and these dogs were distributed evenly throughout both treatment groups (52 evaluable PH 12-treated; 49 evaluable HG Plus-treated). It is important to note that whereas no HW-positive cases were observed in the 52 PH 12-treated dogs from the LMRV region, there were four HW-positive failure cases from the LMRV region in the HG Plus-treated group. For HG Plus, the data represented an overall success rate for the study of 98.2% (214 of 218 dogs negative for HWs across all days); however, there was an 8.2% failure rate (4 HW-positive out of 49) for dogs from the LMRV region alone.

Of the four dogs on HG Plus found to be positive for adult HW infection, three dogs tested positive on Day 365 and one dog on Day 480. While it was not determined if HWs that infected any of these dogs were ML-resistant, these four cases were from areas (three dogs from southern Louisiana and one dog from western Tennessee) where resistant HW strains have been identified previously [18, 19, 22, 23]. All four HG Plus dogs that developed HW infections started on the study (Day 0) between April and May of 2015. For the dogs to have developed infections that were detectable on Day 365 (April to May 2016) by either adult antigen methods and/or microfilaria testing (i.e. at least six-month-old infections required), theoretically they would have needed to have been infected no later than November 2015. Thus, Day 365 testing assessed exposure of dogs from the beginning of the study (April to May 2015) until around six months prior to Day 365 (i.e. November 2015). This period would have included the period of maximum HW transmission (summer months) in the southeastern USA, as determined previously by McTier et al. [49]. Including this season of maximum transmission in the study design ensured enrolled dogs were exposed to the most diverse range of strains of HW possible in a natural situation, including those potentially resistant to MLs.

This unexpected lack of preventive effectiveness for HG Plus in the LMRV is unlikely to be due to compliance failure, as compliance was rigorously documented in this study. Dosing records indicate that the owners of all four dogs were provided the correct dose and quantity of HG Plus and that appropriate doses were administered at the scheduled times, with doses being recalculated regularly based on recent body weight of each dog. Exact doses of ivermectin administered were calculated for each dog after they were removed from the study, and the four dogs received a range of 6.2 to 11.4 µg/kg of ivermectin across all doses. Additionally, these four HW-positive cases for HG Plus appear to be the first ever reported product failures in dogs treated with a ML preventive directly attributed to LOE of a positive control product (not related to issues with potential owner non-compliance [51, 52]) for dogs enrolled in a clinical field study for a new product approval. In contrast to HG Plus, PH 12 was 100% effective in preventing HW disease, even in the 52 dogs from the LMRV). Analysis of the overall data indicated that PH 12 was statistically significantly (*P *= 0.0367) better than HG Plus in preventing heartworm disease in this field study. This field study supports the robust activity of PH 12 in preventing heartworm disease against a range of different heartworm strains existing within the USA.

## Conclusions

A single dose of an extended-release injectable moxidectin suspension (ProHeart ®12) administered at 0.5 mg/kg was well-tolerated and provided 100% prevention of HW disease in dogs for 12 months in both laboratory studies and in natural exposure field studies in the USA. The use of ProHeart® 12 allows veterinarians to ensure correct HW preventive administration, while providing the convenience of a single annual clinic visit/treatment for the owner. ProHeart® 12 offers the opportunity for improved, even complete, compliance with regular use, resulting in consistent HW protection. These attributes could aid in reversing climbing HW disease incidence rates and potentially provide improved clinical outcomes for all dogs, including those exposed to ML-resistant strains of HW.

## Data Availability

All relevant data supporting the conclusions of this article are included within the article.

## Abbreviations

AHS: American Heartworm Society
CVM: US Food and Drug Administration, Center for Veterinary Medicine
GCFL: *Dirofilaria immitis* strain GCF L-01-2014
HG: Heartgard®
HW: heartworm
L_3_: third-stage larvae
LMRV: lower Mississippi River valley
LOE: lack of efficacy
ML: macrocyclic lactone
PH: ProHeart®
PI: post-inoculation
ZoeAL: *Dirofilaria immitis* strain ZoeAL-01-2015
